# Prediction of factors affecting activation of soil erosion by mathematical modeling at pedon scale under laboratory conditions

**DOI:** 10.1038/s41598-020-76926-1

**Published:** 2020-11-19

**Authors:** Saeed Shojaei, Zahra Kalantari, Jesús Rodrigo-Comino

**Affiliations:** 1 Department of Management of Arid and Desert Regions, College of Natural Resources and Desert, Yazd University, Yazd, Iran; 2 Department of Physical Geography and Bolin Center for Climate Research, Stockholm University, 10691 Stockholm, Sweden; 3 Navarino Environmental Observatory, 24001 Messinia, Greece; 4 Department of Sustainable Development, Environmental Science and Engineering, Sustainability Assessment and Management, KTH Royal Institute of Technology, SE-100 44 Stockholm, Sweden; 5 Soil Erosion and Degradation Research Group, Department of Geography, University of Valencia, 46010 Valencia, Spain; 6 Department of Physical Geography, University of Trier, 54296 Trier, Germany

**Keywords:** Environmental sciences, Environmental social sciences, Hydrology, Limnology, Natural hazards

## Abstract

Soil degradation due to erosion is a significant worldwide problem at different spatial (from pedon to watershed) and temporal scales. All stages and factors in the erosion process must be detected and evaluated to reduce this environmental issue and protect existing fertile soils and natural ecosystems. Laboratory studies using rainfall simulators allow single factors and interactive effects to be investigated under controlled conditions during extreme rainfall events. In this study, three main factors (rainfall intensity, inclination, and rainfall duration) were assessed to obtain empirical data for modeling water erosion during single rainfall events. Each factor was divided into three levels (− 1, 0, + 1), which were applied in different combinations using a rainfall simulator on beds (6 × 1 m) filled with soil from a study plot located in the arid Sistan region, Iran. The rainfall duration levels tested were 3, 5, and 7 min, the rainfall intensity levels were 30, 60, and 90 mm/h, and the inclination levels were 5, 15, and 25%. The results showed that the highest rainfall intensity tested (90 mm/h) for the longest duration (7 min) caused the highest runoff (62 mm^3^/s) and soil loss (1580 g/m^2^/h). Based on the empirical results, a quadratic function was the best mathematical model (R^2^ = 0.90) for predicting runoff (Q) and soil loss. Single-factor analysis revealed that rainfall intensity was more influential for runoff production than changes in time and inclination, while rainfall duration was the most influential single factor for soil loss. Modeling and three-dimensional depictions of the data revealed that sediment production was high and runoff production lower at the beginning of the experiment, but this trend was reversed over time as the soil became saturated. These results indicate that avoiding the initial stage of erosion is critical, so all soil protection measures should be taken to reduce the impact at this stage. The final stages of erosion appeared too complicated to be modeled, because different factors showed differing effects on erosion.

## Introduction

The United Nations has formulated 17 overarching sustainable development goals (No Poverty, Zero Hunger, Good Health and Well-Being, Quality Education, Gender Equality, Clean Water and Sanitation, Affordable and Clean Energy, Decent Work and Economic Growth, Industry, Innovation, and Infrastructure, Reduced Inequalities, Sustainable Cities and Communities, Responsible Consumption and Production, Climate Action, Life Below Water, Life on Land, Peace, Justice and Strong Institutions and Partnerships.) for 2030^1^. These include an ambition to combat desertification and reduce land degradation due to different processes such as soil erosion^2–7^. Arid and semi-arid areas are among the most vulnerable areas to soil erosion^7,8^. It is well known that soils are an essential part of the global ecosystem and vital for the survival of human life^9–12^. Humans and natural organisms depend on fertile soils for food production, bio-cycling, plant growth, water, carbon and nutrient storage, material decomposition, etc.^13,14^.

Researchers have accurately identified different forms of soil erosion that can occur under different environmental conditions, due to rain, wind, human, or animal influences^15–19^ or caused by morphological and hydrogeological factors^20,21^. Soil erosion induced by human activities, such as agriculture, could be minimized if correct monitoring and assessment were carried out to develop efficient preventive measures. Natural erosion also depends on uncontrollable factors, but it is not necessary to prevent or mitigate this form of erosion^22,23^. Therefore, understanding the nature of erosion induced by human activities is key to conserving ecosystem services^24,25^.

Studies show that soil erosion by running water mainly depends on land inclination, rainfall intensity, and rainfall duration^23,26^ and, logically, on soil properties and land management^27,28^. Under the impact of raindrops and running water on the surface, soil particles are removed from fertile soil and transported away at a rate which depends on the slope of the land and the amount of runoff^23^. However, it is difficult to predict and determine the point at which this process, which is affected by soil saturation or infiltration, could be minimized or stopped^29–32^.

In research into soil erosion, laboratory and plot-scale rainfall simulation experiments allow the different stages of the water erosion process and the influence of single or combined factors to be investigated under controlled conditions^33,34^. The results of such studies can thus help understand the different spatial–temporal patterns of the main factors involved, knowledge required to develop specific local soil conservation strategies^35,36^. For example, recent studies show that inclination plays an important role in the spread of erosion, including spray, furrow, sheet, or mass erosion^37,38^. Kiani-Harchegani et al.^39^ found higher erosion rates under low rainfall intensity on a slope of 25% compared with 15%, with this increase being directly related to the hillslope inclination. A laboratory study conducted in Italy demonstrated that with increasing inclination and rainfall intensity (30, 60, and 120 mm/h), the bed load decreased and the amount of runoff increased, determining the sediment production boundary^40^. A study examining the effect of hillslope inclination on runoff and sediment production showed that with a more gradual inclination, runoff first decreased and then increased, but the amount of sediment decreased consistently^41^. Studies applying a rainfall intensity of around 47 mm/h on different inclinations (3, 6, and 9%) found that the amount of soil loss showed an inverse negative relationship with inclination^42,43^. In a study in China, researchers found that the rate at which soil particles separated and produced sediment increased at high rainfall intensities, while transfer of soil particles was smallest at low intensities^44^.

In a study in which soils with particles less than 2 and 4.75 mm diameter on different inclinations (0.5, 2.5, 5, 10, and 20%) were subjected to simulated rainfall at an intensity of about 75–80 mm for one hour, the amount of sediment was found to be highly dependent on the slope in unstable conditions, but not in stable conditions^45^. A study investigating the effect of three intensities of rainfall (35, 65, and 95 mm/h) on agricultural soils cropped with rice found that runoff and sediment were highly affected by the vegetation cover, increasing by around 300% with bare soils^46^.

Overall, however, there have been few studies in which the effects of all three factors (inclination, rainfall intensity, and rainfall duration) have been studied simultaneously, in order to identify single-factor effects or multiple interactions and detect when control measures must be taken. The main aim of this study was therefore to investigate the effect of these three factors, alone and in combination, on the amount of soil loss and runoff produced. To achieve this goal, the main factors were each divided into three levels (− 1, 0, + 1), which were applied in different combinations using a rainfall simulator on beds (6 × 1 m) filled with soil from an experimental plot located in the arid Sistan region, Iran. A mathematical model describing the effects of slope, rainfall intensity, and rainfall duration on soil loss and runoff production was then developed. The starting hypothesis was that a model based on accurate data on inclination, rainfall intensity, and rainfall duration can accurately predict the different stages in erosion, information which could be used to develop effective control measures.

## Materials and methods

### Soil sampling and rainfall simulation experiments

**Figure 1:**
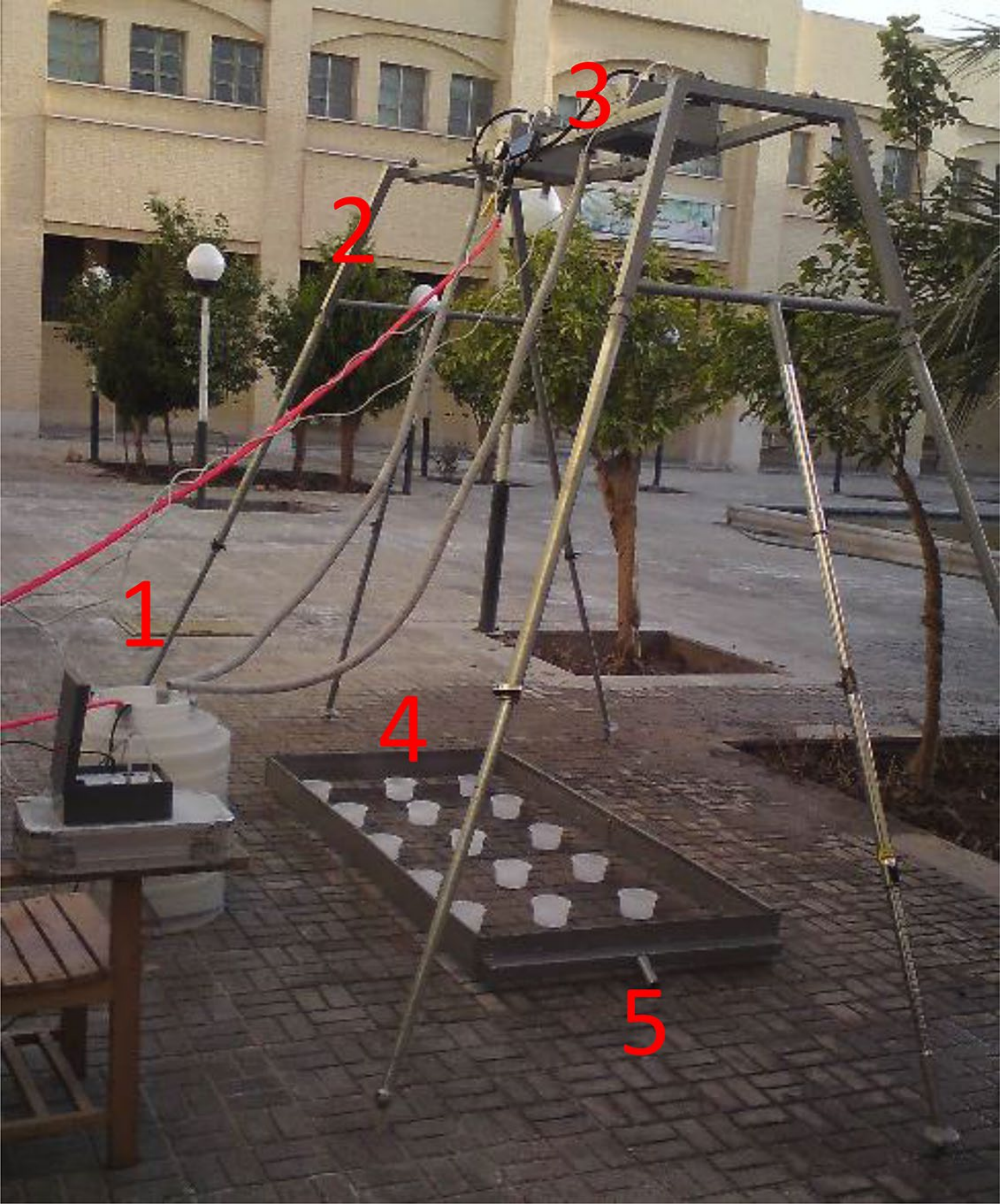
Rainfall simulator used for each test bed. (1) Water tank. (2) Pressure gauge. (3) Micro sprinkler nozzles. (4) Overflow pipe. (5) Measuring cup at the outlet pipe.

Soil samples for the laboratory experiments were collected from the topsoil layer (0–30 cm) in an alluvial area on the Sistan plain in south-east Iran (30°51′ N; 61°01′ E), a region with an arid climate. The soil samples were transported to the Zahedan University Laboratory, Iran, where they were dried in open-air conditions for 24 h. Plant debris and pebbles were then removed, and the entire mass of soil was passed through an 8 mm sieve. A set of test beds, each measuring 6 m × 1 m, was established outdoors (Fig. 1). A drainage layer of mineral pumice was placed in the bottom of each bed, and then 10 cm of soil were poured on the top and pressed down lightly. The erosion-influencing factors tested (rainfall intensity, inclination, and rainfall duration) at the different levels (− 1, 0, + 1) were applied to these beds, with artificial rainfall using a rainfall simulator, supplying distilled water with pH 7 (Fig. 1). The three levels of rainfall duration tested were 3, 5, and 7 min, the rainfall intensity levels were 30, 60, and 90 mm/h, and the inclination levels were 5, 15, and 25%. The slope was achieved by setting the base of the bed at the desired inclination before adding soil. Before conducting each experiment, non-erosive precipitation of 5 mm/h was applied to prepare the test beds and restore the soil to its natural moisture content^47^. The average rainfall amount in the different intensity and duration treatments was 1.5 mm, the speed of application was 6.8 m/s, and the power was 0.40 W/m^2^. The average runoff power was 0.035 W/m^2^.

### Measurement of runoff and sediment

Sheet flow runoff at the outlet of the beds during the simulated rainfall events was measured at intervals of one minute, with three replicates of 17 runs. Each test included different combinations of the different factor levels (Table 1). The amount of soil loss produced in each test was collected in a single container (Fig. 1). The dry mass of soil in the container was determined by oven-drying at 105 °C for 24 h. Based on the amount of soil loss produced in each rainfall event (3, 5, or 7 min) and plot area (1.6 m^2^), soil loss per minute was calculated for each intensity of precipitation tested.

**Table 1:** Different levels tested, units, and codes of the three variables assessed in the laboratory experiment.

Variable	Units	Factor code	Level		
			− 1	0	1
Rainfall intensity	mm/h	A	30	60	90
Rainfall duration	min	B	3	5	7
Inclination	%	C	5	15	25

### Laboratory tests and data treatment

The levels selected for the three rainfall durations (factor A), rainfall intensity levels (factor B), and inclination levels (factor C) in the experiment are shown in Table 1. These levels were chosen based on Eq. (1). Runoff volume and soil loss rate were simulated and predicted using the Design-Expert software 10.0.8^48–51^. This software is able to assess the necessary inputs and outputs of a theoretical process in a specific procedure to obtain the most accurate results, in order to identify the optimal process or product. This procedure is useful to reduce time costs and expenses.

1 $${x}_{i}=\frac{{x}_{i-}{x}_{0}}{\Delta {x}_{i}}$$

X_i_ = Independent encoded variable value, X_0_ = Center point of actual values of the variable, ΔXi = Change in value.

Analysis of variance (ANOVA) was applied to the runoff and erosion data obtained. Based on the *P* values (Table 2), the importance of the variables was evaluated and an appropriate mathematical model was developed (Eq. 2). Signal-to-noise ratio was applied to test the accuracy of the mathematical models, with values greater than 4 taken to denote good accuracy^52^.

2 $$Y = b_{0} + \sum\limits_{i = 1}^{4} {b_{i} x_{i} + \sum\limits_{i = 1}^{4} {b_{ii} x_{i}^{2} + \sum\limits_{i = 1}^{3} {\sum\limits_{j = i + 1}^{4} {b_{ij} x_{i} x_{j} } } } }$$

**Table 2:** Statistical parameters obtained for the model.

Source		Sum of squares	Degrees of freedom	Mean square	F-value	Prob > F	Remarks
Runoff	Quadratic	616.25	3	205.42	12.86	0.0031	Suggested
	Pure Error	12.80	4	3.20	1.05	0.4045	
Sediment	Quadratic	1.842	3	61,400.00	12.90	0.0031	Suggested
	Pure Error	0.000	4	0.000	5.74	0.0100	

Y = predicted answer, β_0_ = y-intercept coefficient, β_i_ = linear coefficient of the parameters, β_ij_ = secondary interaction coefficient, b_ij_ = second order coefficient, x_i_, x_j_ = independent encoded variables.

## Results and Discussion

### Soil erosion results (experimental and predicted)

Analysis of variance and factor values for the erosion data showed that the calculated F-value value (11.59) was higher than the observed value (Table 3). The R^2^ value was 0.94 and the Adj-R^2^ was 0.85, showing good accuracy of the model (Eq. 2) in predicting and describing soil erosion. The signal-to-noise ratio obtained was 9.766, also confirming good accuracy of the erosion prediction model.

**Table 3:** Analysis of variance (ANOVA) results obtained for runoff (Q, mm^3^/s) and erosion (soil loss, g/m^2^/h).

Source	Sum of squares	DF	Mean square	F-value	*P* value	Remarks
*Runoff*						
Model	9806.71	9	1089.63	12.13	0.0017	Significant
Rainfall intensity	1653.12	1	1653.12	18.40	0.0036	
Rainfall duration	66.12	1	66.12	0.74	0.4194	
Inclination	312.50	1	312.50	3.48	0.1045	
AB	900.00	1	900.00	10.02	0.0158	
AC	756.25	1	756.25	8.42	0.0230	
BC	2652.25	1	2652.25	29.51	0.0010	
A^2	2019.41	1	2019.41	22.47	0.0021	
B^2	297.09	1	297.09	3.31	0.1119	
C^2	843.04	1	843.04	9.38	0.0182	
Residual	629.05	7	89.86			
Lack of Fit	616.25	3	205.42	64.19	0.0008	Significant
*Sediment loss*						
Model	2.745	9	3.050	11.59	0.0020	Significant
Rainfall intensity	12,800.00	1	12,800.00	0.49	0.5080	
Rainfall duration	1.656	1	1.656	62.94	< 0.0001	
Inclination	0.000	1	0.000	0.000	1.0000	
AB	57,600.00	1	57,600.00	2.19	0.1825	
AC	0.000	1	0.000	0.000	1.0000	
BC	0.000	1	0.000	0.000	1.0000	
A^2	88,526.32	1	88,526.32	3.36	0.1093	
B^2	8.717	1	8.717	33.13	0.0007	
C^2	8526.32	1	8526.32	0.32	0.5870	
Residual	1.842	7	26,314.29			
Lack of Fit	1.842	3	61,400.00			

*Runoff* ${R}^{2}$= 0.9397, Adj-${R}^{2}$= 0.8622, signal to noise ratio = 10.591.

*Sediment loss* ${R}^{2}$= 0.9371, Adj-${R}^{2}$ = 0.8563, signal to noise ratio = 9.766.

**Figure 2:**
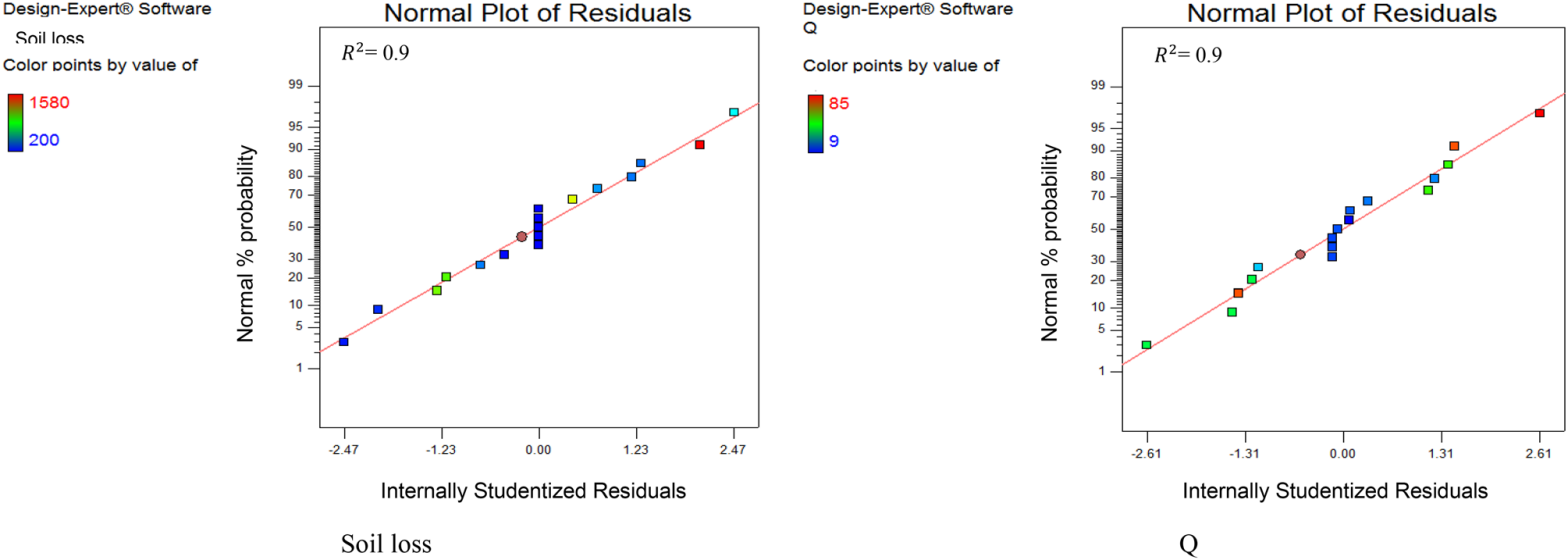
Scatter plot obtained for (left) sediment production and (right) runoff (Q) production. *Corrected to 5%.

The scatter plots (Fig. 2) for runoff and soil loss showed that the real and predicted data were close to each other, which confirmed the accuracy of the model in predicting the amount of sediment and runoff (Table 4).

**Table 4:** Experimental and predicted results obtained for production of runoff (Q, mm^3^/s) and erosion (soil loss, g/m^2^/h) in runs 1–17 of the experiment testing different levels (− 1, 0, + 1) of rainfall intensity, rainfall duration, and inclination (see Table 1).

Run	Rainfall	Rainfall	Inclination	Q (mm^3^/s)		Sediment (g/m^2^/h)	
	intensity	duration		Experimental	Predicted	Experimental	Predicted
1	0	0	0	14	15.20	200	200
2	0	− 1	1	42	54.38	210	245
3	0	1	1	9	8.63	1050	1155
4	0	1	− 1	85	72.63	1190	1155
5	0	0	0	16	15.20	200	200
6	1	− 1	0	79	72.00	260	425
7	0	0	0	18	15.20	200	200
8	0	0	0	14	15.20	200	200
9	− 1	0	− 1	24	29.38	230	430
10	− 1	0	1	51	44.38	370	430
11	0	− 1	− 1	15	15.38	350	245
12	0	0	0	14	15.20	200	200
13	− 1	− 1	0	19	13.25	360	265
14	1	0	− 1	79	85.63	410	350
15	− 1	1	0	42	49.00	1580	1415
16	1	1	0	42	47.75	1000	1095
17	1	0	1	51	45.63	550	350

The final models obtained for runoff and soil loss production in the simulated rainfall experiments are shown in Eqs. (3) and (4), respectively.

3 $${\text{Runoff }} = + {15}.{2}0 + {14}.{\text{38A}} + {2}.{\text{87B}} - {6}.{\text{25C}} - {15}.00{\text{AB}} - {13}.{\text{75AC}} - {25}.{\text{75BC}} + {25}.{9}0{\text{A}}^{{2}} + {8}.{4}0{\text{B}}^{{2}} + {14}.{\text{15C}}^{{2}}$$

4 $${\text{Soil}}\;{\text{loss}} = + {2}00.0 - {4}0.00{\text{A}} + {455}.00{\text{B}} + 0.00{\text{C12}}0.00{\text{AB}} + 0.00{\text{AC}} + 0.00{\text{BC}} + {145}.00{\text{A}}^{{2}} + {455}.00{\text{B}}^{{2}} + {45}.00{\text{C}}^{{2}}$$

where *A* is rainfall intensity (mm/h), *B* is rainfall duration (min), and *C* is soil loss (%).

Analysis of variance and factor values obtained from runoff data showed that the calculated F-value (12.32) was higher than the observed value. The R^2^ value was high (0.9397), showing that the model described the empirical data and predicted erosion with high accuracy. The value of Adj-R^2^ was also high (0.8622), confirming the accuracy of the model in evaluating the amount of runoff. The signal-to-noise ratio was 10.591, providing further confirmation of model accuracy^49^.

### Single-factor analysis of changes in runoff production

**Figure 3:**
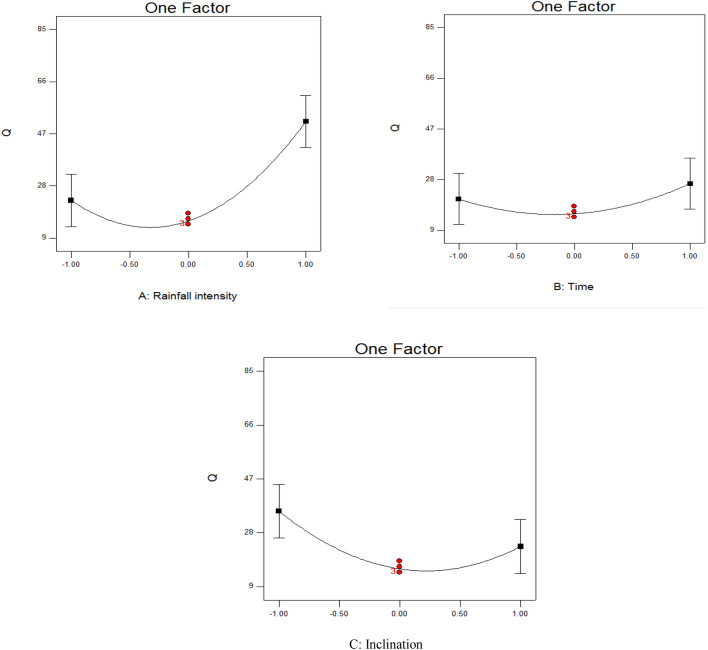
Results of single-factor analysis of the effect of changes in (**a**) rainfall intensity (30, 60, and 90 mm/h), (**b**) rainfall duration (3, 5, and 7 min), and (**c**) inclination (5, 15, and 25%) on runoff (Q, mm^3^/s) from the test beds.

The analysis of single-factor effects showed that the amount of runoff was much more strongly affected by changes in runoff intensity than by changes in rainfall duration and inclination (Fig. 3). The amount of runoff increased exponentially with increasing rainfall intensity related to the highest intensity tested (90 mm/h). This is because with increasing intensity of rainfall, a greater amount of rainfall reaches the soil surface per unit time and there is less time for it to penetrate the soil, so it runs off the surface. Changes in rainfall duration resulted in few changes in the amount of runoff, with runoff increasing only at the end of the experiment due to the continued rainfall leading to soil saturation.

Runoff volume decreased with an increasing inclination between 5 and 15%. The reason for this relates to the speed of water flow, i.e., at high slopes runoff is rapidly removed and the thickness of the runoff layer is reduced. Due to the presence of water particles moving at high intensity, the rate of penetration into steeper slopes also increases. When the inclination was increased further to 25%, the amount of runoff began to increase again (Fig. 3). However, a study on a Mollisol in China examining runoff changes due to differences in slope (5 and 10°), with rainfall intensity of 0 and 70 mm/h (RI0, RI70), found that runoff decreased in the order rainfall intensity 70 + inflow rate 70 > rainfall intensity 70 + inflow rate 0 > rainfall intensity 0 + inflow rate 70, and that increasing the slope from 5 to 10 increased the runoff amount by 2.5- to 6.9-fold^53^.

### Interactive effects of multiple factors on runoff production

**Figure 4:**
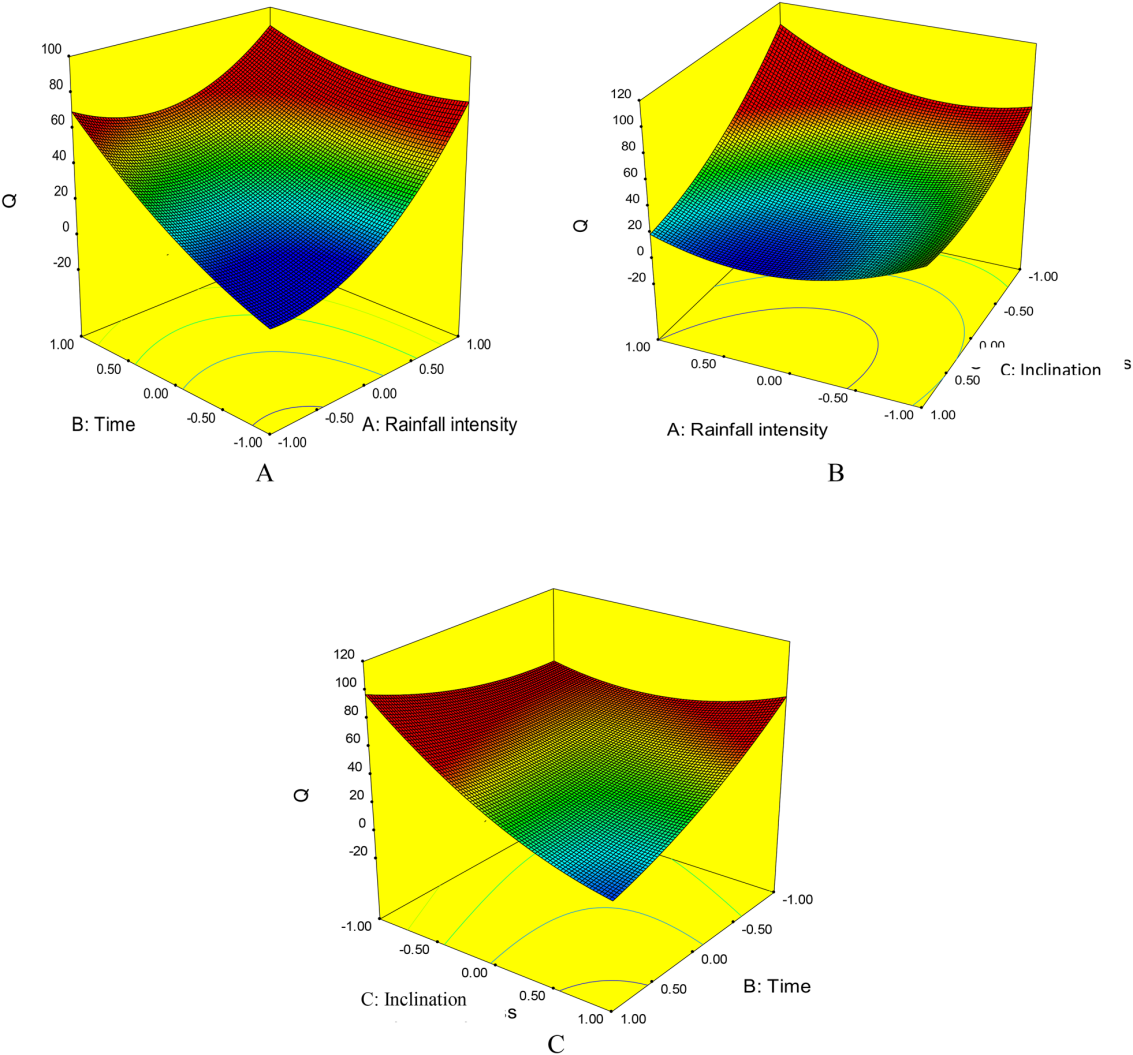
Comparison of the combined effect of (**A**) rainfall intensity and rainfall duration, (**B**) rainfall intensity and slope, and (**C**) rainfall duration and slope on runoff (Q, mm^3^/s) from the test beds.

Three-dimensional diagrams were used to represent the simultaneous effect of different factors on the amount of runoff generated by the simulated rainfall experiments (Fig. 4). The diagrams obtained showed that as rainfall intensity increased, the amount of runoff also increased within a shorter time, because of a faster decrease in the amount of water infiltration. An increase in rainfall intensity had the greatest effect in increasing runoff and was most influential in runoff production, giving an exponential increase in runoff production over time (Fig. 4A).

Comparison of the combined effect of inclination and rainfall intensity showed that increasing rainfall intensity produced more runoff than steeper slope. However, with increasing inclination combined with increasing intensity of rainfall, the amount of runoff increased under all conditions (Fig. 4B). Comparison of the combined effect of inclination and rainfall duration showed that both factors had a similar effect in increasing runoff. With increasing time and increasing slope, the amount of runoff increased linearly (Fig. 4C).

The formation of runoff depends on multiple factors apart from the three main factors examined in this study, such as soil moisture, soil texture and structure, soil surface characteristics, etc.^54–56^. For a given set of soil and site conditions, higher-intensity rainfall can increase runoff and sediment production by more than 30–50% compared with low-intensity rainfall, and this trend increases with increasing rainfall intensity^57^. For example, a study investigating runoff production from agricultural soils found that increasing the rainfall intensity from 35 to 95 mm/h increased runoff production by around threefold, but that soil surface characteristics could be effective in reducing erosion^46^. Production of runoff directly affects the production of sediment, e.g., a change of slope from 5 to 25% at a rainfall intensity of 90 mm/h has been found to increase the amount of runoff by fourfold and the amount of sediment by fivefold^39^.

### Single-factor analysis of changes in soil loss

**Figure 5:**
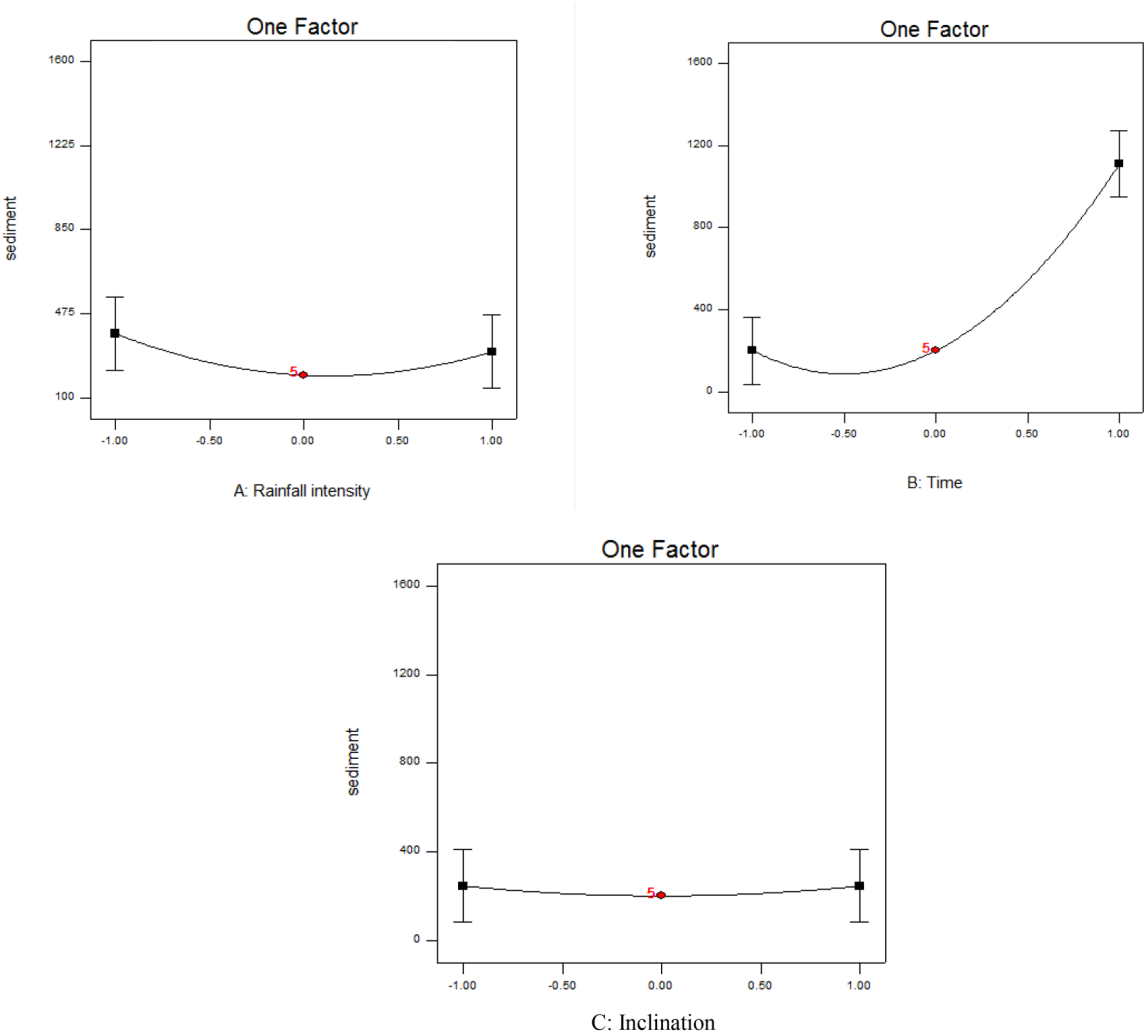
Results of single-factor analysis of the effect of changes in (**A**) rainfall intensity (30, 60, and 90 mm/h), (**B**) rainfall duration (3, 5, and 7 min), and (**C**) slope (5, 15, and 25%) on the amount of sediment (g/m^2^/h) lost from the test beds.

The analysis of single-factor effects showed that the amount of soil loss produced was much more strongly affected by increasing rainfall duration than by changes in rainfall intensity and inclination (Fig. 5). Runoff amount increased exponentially over time, to reach a maximum at a rainfall duration of 7 min, because of a greater volume of sediment entering the sediment trap as rainfall continued. Sediment production was high at the beginning of the experiment, but decreased by the end. Rainfall fluctuations and rainfall intensity directly affect the amount of sediment eroded from soil with a particular texture, while increasing the inclination can increase erosion^58,59^.

In our experiments, increased rainfall intensity first increased, and then decreased, the loss of sediment. This could be because the amount of transportable sediment declined over time, so by the end of each rainfall event there was less to be washed away from the bed surface. With increasing rainfall and increasing diameter of raindrops from less than 0.25 mm to more than 0.25 mm, sediment production has previously been found to increase to 89.7%^60^. The single-factor analysis also showed that soil loss remained almost constant with increasing slope (Fig. 5). The reason for this could be related to the nature of soil particles, e.g., it is possible that the intensities applied were able to transfer particles of a certain size, and slope changes had little effect on the amount transported^61,62^.

### Interactive effects of multiple factors on soil loss

**Figure 6:**
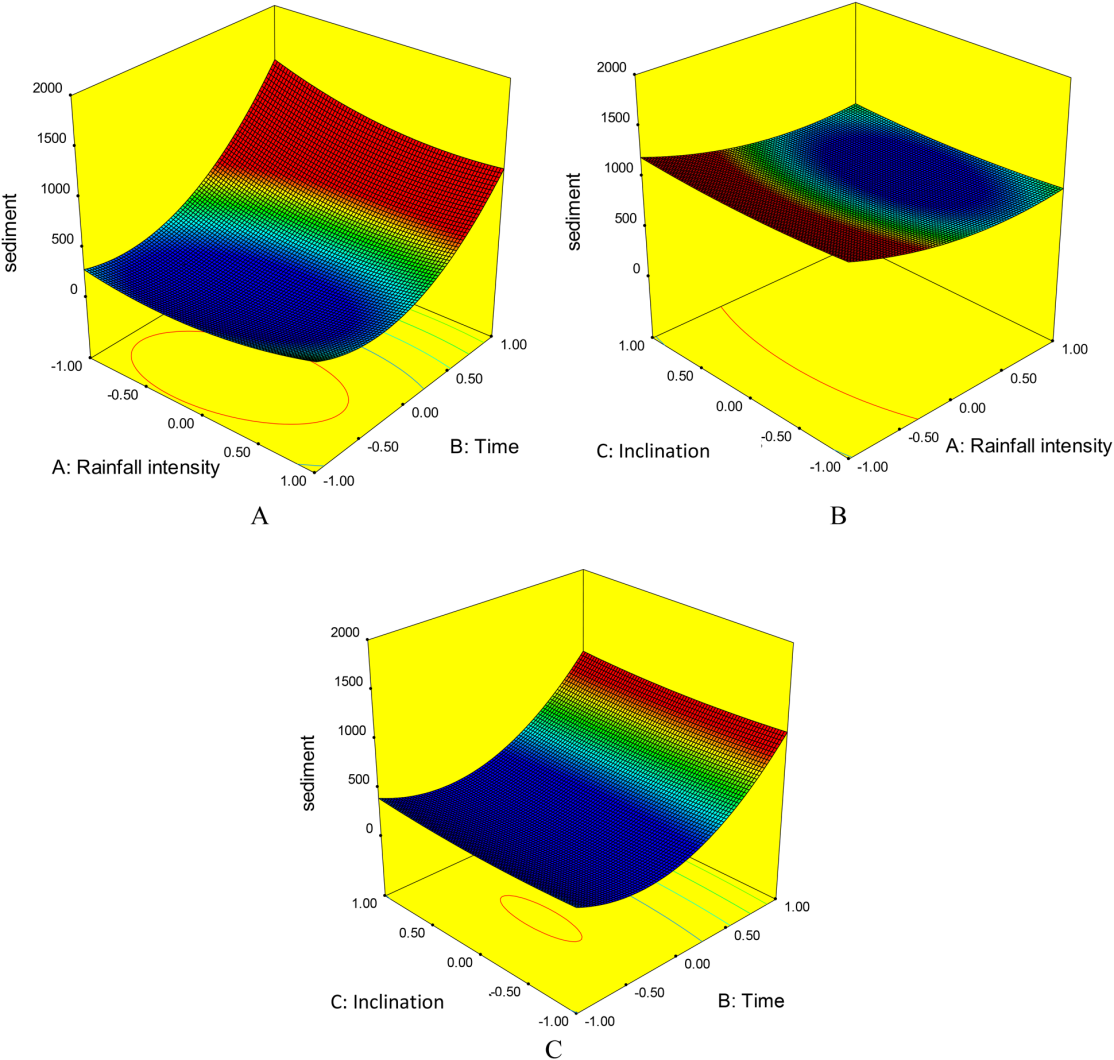
Comparison of the combined effect of (**A**) rainfall intensity and rainfall duration, (**B**) rainfall intensity and slope, and (**C**) rainfall duration and slope on sediment loss (g/m^2^/h) from the test beds.

Three-dimensional diagrams showing the simultaneous effects of the different factors on the amount of sediment produced in the simulated rainfall events revealed that as the duration and intensity of precipitation increased, the amount of sediment also increased (Fig. 6). Rainfall duration had a stronger effect than rainfall intensity, but the effect of both factors was exponential and the amount of sediment increased sharply with increasing test duration (Fig. 6A).

Comparison of the combined and effect of rainfall intensity and inclination on soil loss activation showed that the effect of rainfall intensity was stronger. Higher-intensity rainfall is likely to separate more soil particles and move them more rapidly. Increasing the inclination of the plot surface further increased the production of sediment (Fig. 6B). Comparison of the combined effect of rainfall duration and inclination showed that both these actors were effective in increasing runoff. Again, rainfall duration had a stronger effect, and the increase in sediment over time was exponential (Fig. 6C). The changes in sediment production indicate the complexity of the erosion cycle, which cannot be examined in one dimension. These changes reflect the combined effects of the intensity and duration of precipitation and differences in soil characteristics^42^. Erosion and sediment changes are very complex in the early stages, but over time the process of runoff and sediment alters to constant change (ascending or descending)^63^. The level of change is determined by the soil texture^64–66^, the initial soil moisture^37,67^, and the intensity of precipitation^36,66^. Studying these in an integrated manner was a novel aspect of the present study.

The results of this study showed that sediment loss increased in the first stage of precipitation and with increased rainfall intensity, but decreased over time as the availability of transportable particles declined. However, higher-rainfall intensity increases the capacity of runoff to transport sediment, allowing it to carry other, larger soil particles, as reported in other study areas under field conditions^68–70^. Our results showed that inclination change also affected the amount of sediment production, confirming previous findings^71,72^.

## Conclusions

The effects of three levels of three factors (rainfall intensity, rainfall duration, and inclination) were examined under laboratory conditions with simulated rainfall experiments. The results showed that production of soil loss and runoff, and the course of the erosion process, were altered when several factors were applied simultaneously. Single-factor analysis showed that rainfall intensity was more effective than other factors in runoff production, but that rainfall duration played a more important role in sediment production. Multi-factor analysis showed that the combined effect of rainfall intensity and slope increased runoff production by about twice the level caused by the single most important factor (i.e., rainfall intensity). Moreover, the combined effect of rainfall intensity and duration increased soil loss production by about 24% compared with the most important single-factor (i.e., rainfall duration). The results also showed that in the initial stages of erosion, when there is more transportable sediment available at plot level (soil particles), the amount of runoff was lower and the rate of erosion was higher. In the final stages of erosion, the amount of soil loss decreased due to lack of erosive particles, but a larger volume of runoff was produced from the saturated soil. A quadratic model was found to be the best model for predicting the effects of rain intensity, rainfall duration, and slope on runoff (F-value = 12.86) and sediment (F-value = 12.90). The R^2^ and Adj-R^2^ values were high in both cases (0.94 and 0.86, respectively), indicating high accuracy of model prediction based on experimental data. Water erosion removes considerable volumes of soil in different regions worldwide. This study provides a more accurate method for assessment of the water erosion process at laboratory and larger scale. The results can be used to formulate appropriate national and international measures to control erosion. They can also be used in designing water structures, and in assessing soil sensitivity to erosion and the effect of different factors on water erosion.
